# Supplementary material for: Nanoval Technology—An Intermediate Process between Meltblown and Spunbond
Source: Materials (Basel). 2023 Apr 6;16(7):2932. doi: 10.3390/ma16072932 (PMC10096243; doi:10.3390/ma16072932)
Supplement: Supplementary file 1 [file materials-16-02932-s001.zip › materials-2295489-supplementary.pdf]

Supplementary Material to

Flexible creation of fine and coarse fiber diameters in meltspun nonwovens using the Nanoval Laval-die process as an intermediate technology between meltblown and spunbond processes

T. Höhnemann*, J. Schnebele*, I. Windschiegl, W. Arne

Material characterization and suitability characterization for the Nanoval process

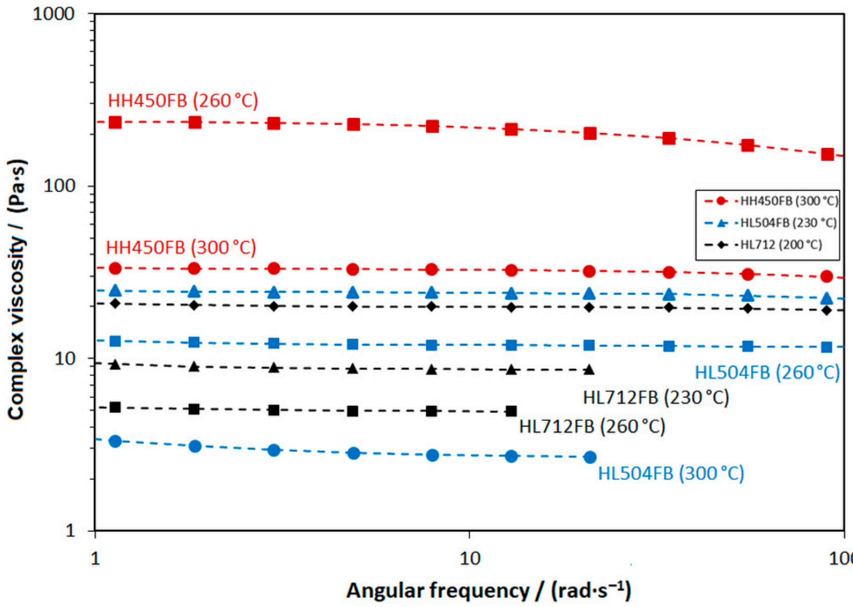

Figure S1: P*lot of complex viscosity vs. shear rate* (ε = 10%) *for the different polypropylenes at different temperatures.*

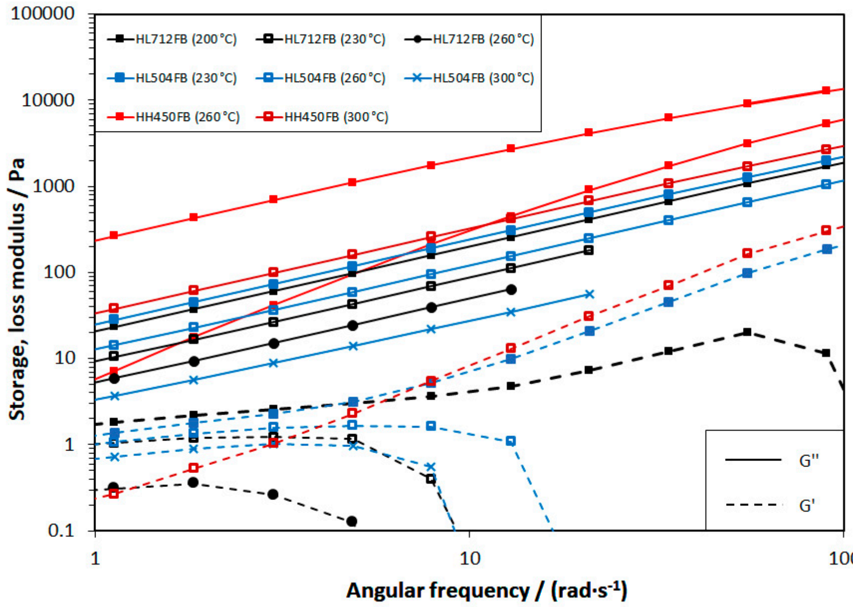

Figure S2*:* Plots of storage and loss modulus from the frequency sweeps (ε = 10 %) for *for the different polypropylenes at different temperatures.*

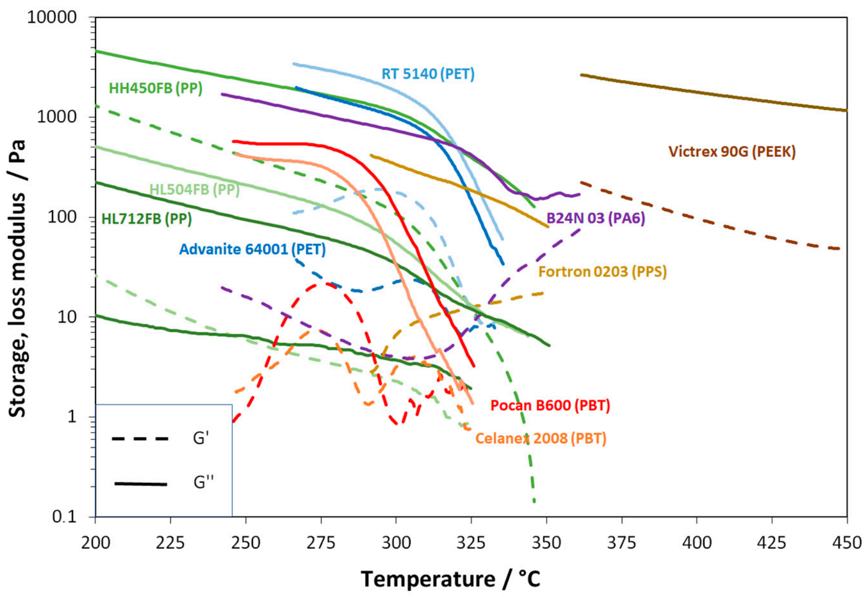

Figure S3*:* Plots of storage and loss modulus from the temperature sweeps (ε = 10 %, ω = 10 rad·s^{-1}, $\dot{T}$ = 0.5 K·min^{-1}) for all polymers tested.

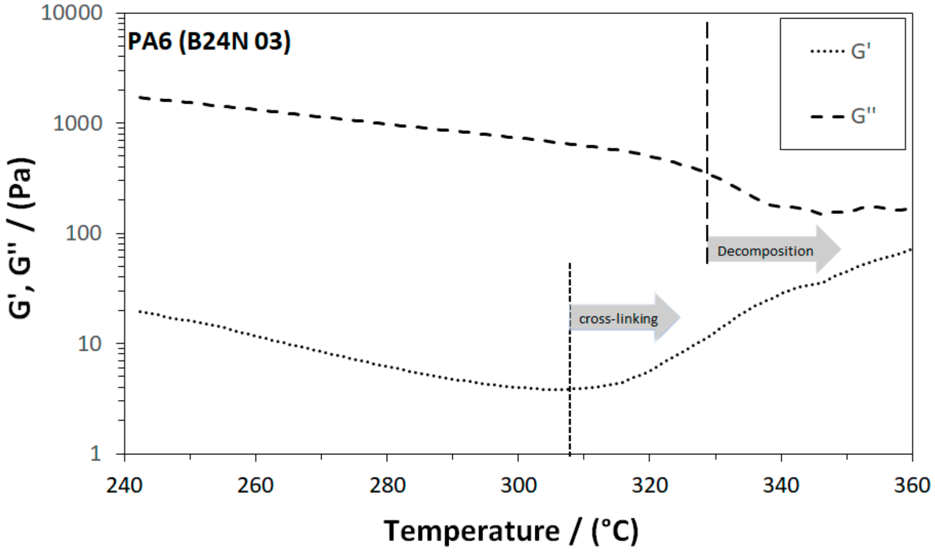

Figure S4: *Indication for interaction of degradation and crosslinking of B24NO3 (PA6) in the plot of G'/G'' vs. temperature.*

Experimental characterization of the Nanoval process

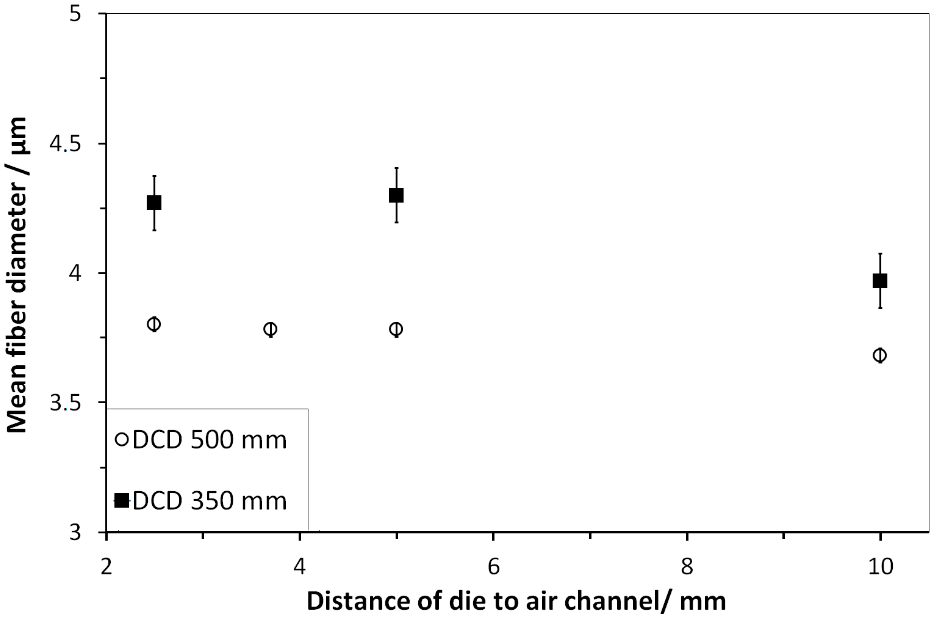

Figure S5: *Plot of mean fiber diameter vs. distance of die to air channel (DDAC) for two different DCDs (PP HL712FB, $\dot{V}$ = 3.8 g.ho-1.min-1, $\dot{V}_{air}$ = 220 Nm^{3}·h^{-1})*

Table S1: *Resulting fiber diameters of polypropylenes of different molar mass at same process setting; T_{melt} = 300 °C, $\dot{V}_{air}$ = 220 Nm^{3}·h^{-1}.*

**Polymer**	**Median**	**Mean**	**stdev.**	**Die pressure**
-	Mm	µm	Mm	bar
$\dot{V}$ = 1.9 g·ho^{-1}·min^{-1}:				
PP HL 712 FB	2.94	3.74	0.17	25.4
PP HL 504 FB	5.47	6.78	0.41	35.5
$\dot{V}$ = 3.8 g·ho^{-1}·min^{-1}:				
PP HL 712 FB	2.09	2.43	0.13	20.0
PP HL 504 FB	3.87	4.66	0.37	27.5

The higher viscosity of the polymer melt passing the die results in an increase of the die pressure drop of around 40% and results in almost doubled fiber diameters. So, the identic air amount is not sufficient to stretch the higher viscous polymer mass as efficient and less homogeneously.
